# Molecular and Cellular Mechanisms of KSHV Oncogenesis of Kaposi's Sarcoma Associated with HIV/AIDS

**DOI:** 10.1371/journal.ppat.1004154

**Published:** 2014-07-10

**Authors:** Lucas E. Cavallin, Pascal Goldschmidt-Clermont, Enrique A. Mesri

**Affiliations:** 1 AIDS Malignancies Scientific Working Group, Miami Center for AIDS Research, Department and Graduate Program of Microbiology and Immunology, University of Miami Miller School of Medicine, Miami, Florida, United States of America; 2 Viral Oncology Program, Sylvester Comprehensive Cancer Center, University of Miami Miller School of Medicine, Miami, Florida, United States of America; University of Florida, United States of America

## AIDS-Associated Kaposi's Sarcoma: An Oncogenic Consequence of Infection with KSHV and HIV

Twenty years after its discovery [1], Kaposi's sarcoma herpesvirus (KSHV) or human herpesvirus-8 (HHV-8) continues to be an enigmatic oncovirus, while AIDS-associated Kaposi's sarcoma (AIDS-KS) remains a clinical challenge in endemic regions in Africa and for some patients receiving anti-retroviral therapy (ART) [2]–[4]. KSHV, a gamma-2 herpesvirus, is the etiological agent of Kaposi's sarcoma (KS) [2], [3]: (1) KSHV is strictly associated with all clinical forms of the disease, including classic KS affecting elderly individuals of Mediterranean or Ashkenazi origin, transplant-associated KS, endemic KS affecting sub-Saharan countries, and AIDS-associated or epidemic KS. (2) KSHV is found in KS spindle cells. (3) KSHV infection precedes the onset of KS. (4) KSHV seroprevalence is higher in areas of high KS incidence. (5) KSHV encodes many viral oncogenes. (6) KSHV transforms and induces tumorigenesis in endothelial cell lineage [3], [5], [6]. Like other human oncogenic viruses [7], KSHV infection alone is generally not sufficient to cause KSHV-associated cancers, which also include two B-cell lymphoproliferative disorders associated with HIV/AIDS: Multicentric Castlemans Disease and Primary Effussion Lymphoma [8]. This indicates that other co-factors are necessary for malignant transformation [2], [7]. KSHV seroprevalence in the general population ranges from less than 10% in the United States and Northern Europe to 30%–50% in endemic areas, where KS lifetime-incidence could be as high as 1% [9]. KS incidence increases dramatically in HIV-infected individuals, indicating that HIV/AIDS is a potent co-factor for KSHV oncogenesis [2]–[4], [9]. Yet, even in this high-risk group, the majority of KSHV-infected individuals will not develop KS, indicating that complex interactions between KSHV, genetic susceptibility, immune status, and HIV infection determine the oncogenic outcome of KSHV infection.

## The Cellular Origin of KS: In Search of the Mysterious KS Spindle Cell Progenitor

AIDS-KS lesions are characterized by proliferating KSHV-infected spindle cells, intense angiogenesis with erythrocyte extravasation, and inflammatory cell infiltration [2], [3]. The origin of the spindle cells remains enigmatic, because they express markers of multiple cellular lineages, including endothelial, monocytic, and smooth muscle [2], [3]. Although KSHV infection results in important morphological and transcriptional changes that convey traits of cell transformation, few of these cell types can become transformed and tumorigenic [2], [3]. The heterogeneous expression of cellular markers and the multifocal nature of KS lesions suggests that a circulating hematopoietic progenitor cell (HSC) could give rise to KS spindle cells, especially endothelial progenitor cells (EPCs) and mesenchymal stem cells (MSC), as they both have the capacity to differentiate into endothelial lineage [10]. Evidence pointing to the existence of a circulating KS progenitor, which is increased in AIDS-KS [11], includes: (a) KS spindle cells in renal-transplant recipients are from donor origin [12]. (b) KS exhibits the “Koebner phenomenon,” whereby KS lesions appear at sites of injuries, suggesting that inflammatory cytokines can recruit circulating KS progenitors to the site of trauma [10]. (c) Inflammatory cytokines, elevated in HIV/AIDS, can recruit potential KS progenitors and induce spindle cell differentiation and proliferation [11]. (d) Three KS models made from mouse endothelial lineage HSC cells [6], [13] and one from rat MSC [14] formed tumors in a KSHV-dependent manner, suggesting that these populations contain cell types in which KSHV infection is oncogenic.

Identifying the KS progenitor is complex since KSHV infection causes transcriptional reprogramming and KSHV can infect many cell types due to broad expression of its entry receptors, the α3β5 integrin (ITGA3/ITGB5) and the EphrinA-receptor-2 (EPHA2). Thus, it is difficult to know whether the phenotypic markers of infected KS spindle cells correspond to those of the target cells, or are the consequence of KSHV's ability to reprogram host-cell transcription [6], [10].

## KSHV Encodes an Oncogenic Armamentarium with the Potential to Induce All Malignant Phenotypic Characteristics of KS

KSHV encodes for several viral oncogenes, including 14 host homologues that carry the potential to induce all the malignant phenotypic characteristics—cancer hallmarks—of KS [2], [7]. KSHV can establish either a latent or lytic infection. Latency is an immune-silent state in which KSHV replicates along with the host by expressing a restricted number of genes needed for episomal maintenance [2], [3]. During lytic infection, KSHV expresses the full replication program to produce new virions. Latent gene expression favors viral persistence and replication by promoting host-cell proliferation and survival (reviewed in [2], [3]). Among the latent KSHV genes, LANA was shown to inactivate the p53 and pRB tumor suppressor pathways [15]. The KSHV cyclin homologue (v-cyclin) is able to induce cell cycle entry by counteracting both p21 and p27 Cyclin-dependent kinase (CDK) inhibitors [16]. The viral FLICE-inhibitory protein (vFLIP) can constitutively activate NFkB, promoting cell survival by up-regulating transcription of anti-apoptotic genes such as *BCL-2* and *A20* [17]. Other KSHV latent genes, such as Kaposin (K12) and the KSHV-encoded miRNA [18], have been shown to further collaborate in inducing the KS malignant phenotype.

KSHV lytic expression includes genes that favor viral replication by affecting the DNA damage response, promoting survival, and evading the immune response. As a consequence, lytic genes can induce the following KS malignant phenotypes: (1) Immune evasion [19]: IRF homologues (vIRFs 1–4) can inhibit the IFN response and ORFK4 inhibits the complement system, while K3 and K5 down-regulate immune recognition genes by ubiquitination. (2) Genetic instability: vIRF-1 can obstruct the DNA damage response by inhibiting p53-mediated activation of ATM, and the viral G protein-coupled receptor (vGPCR) can induce oxidative DNA damage via Rac1-mediated activation of ROS [20]. (3) Anti-apoptosis: vGPCR and K1 have been shown to activate the anti-apoptotic NFkB pathway while vIRF-1 was shown to inhibit BH3-only proapoptotic mediators, such as BIM.

KSHV lytic genes may also favor reinfection of KS progenitors by inducing angiogenesis and inflammation that help to recruit un-infected endothelial-lineage and hematopoietic cells. vGPCR, a constitutively active homolog of the Groα and IL-8 receptors CXCR1/2, activates MAPKs, NFkB, and PI3K-AKT-mTOR pathways, leading to an angiogenic switch and endothelial cell immortalization caused by up-regulation of VEGF and its receptor-2 KDR (VEGFR2) [21], [22]. vGPCR induces angiogenic KS-like tumors in transgenic mice by up-regulating VEGF, IL-6, Angiopoietin-2 (ANGPT2), and PDGF [23], [24]. K1 contains an ITAM motif that is able to constitutively induce angiogenic and inflammatory responses via AKT and NFkB [25]. In addition, KSHV encodes several virokines, including a viral IL-6 (vIL-6) that can potently activate gp130 signaling [26] and other viral homologs to angiogenic chemokines (vMIP-I/III or vCCL-I-III).

## KSHV and Oncogenesis of AIDS-KS: A Puzzle at the Crossroads of KSHV Biology, Cellular Cancer Pathways, and HIV/AIDS

**Figure 1:**
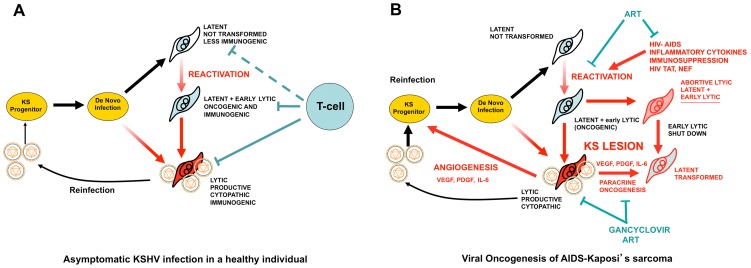
KSHV pathobiology in healthy and HIV/AIDS patients. (A) In a healthy host, KSHV infection of a KS progenitor is not oncogenic, since it leads to latent infection or to cytopathic lytic replication. Reactivation leading to oncogenic lytic gene expression is under immunological control. (B) Scenario for AIDS-KS pathogenesis according to the Paracrine Oncogenesis and Abortive Lytic Hypotheses. In HIV/AIDS, decreased immunosurveilance, inflammatory cytokines and HIV Tat lead to KSHV reactivation and reinfection. This leads to increased, uncontrolled, early lytic oncogenic gene expression, with concomitant risk of cell transformation by somatic host cell oncogenic alterations. Upon transformation, cells shut down early lytic oncogenes. Latently infected transformed cells are stimulated in a paracrine manner by angiogenic and proliferative factors released from lytically infected or abortive lytic cells (paracrine oncogenesis, see details in Figure 2). In addition, lytically infected cells provide a constant source of virions for reinfection, while angiogenesis and inflammation recruit target KS progenitors. ART inhibits KSHV reactivation and lytic replication through immune reconstitution and decreased levels of HIV viral loads. Gancyclovir inhibits viral replication and lytic gene expression.

**Figure 2:**
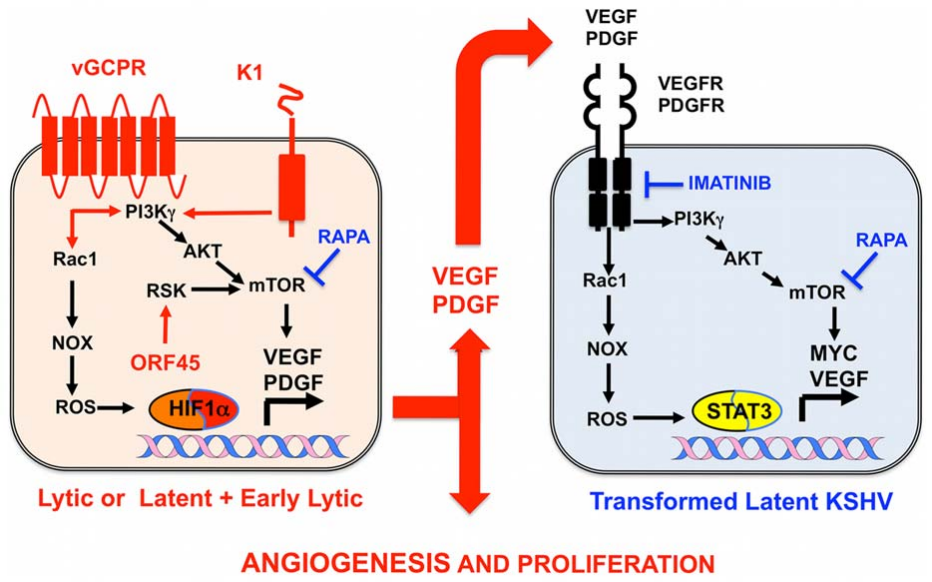
Molecular mechanisms, therapeutic targets, and clinically tested drugs in AIDS-KS paracrine viral oncogenesis. In KS spindle cells lytically infected with KSHV cells or latently infected spindle cells expressing early lytic genes, KSHV genes such vGPCR, K1, and ORF45 constitutively trigger signaling cascades, leading to mTOR and ROS activation, which induce transcription and translation of PDGF and VEGF. These secreted growth factors can act in a paracrine manner to activate the same signaling cascades in latently infected cells expressing VEGF and PDGF receptors to drive KS cell proliferation and angiogenesis. Rapamycin (RAPA), which inhibits mTOR, and Imatinib (IMA), which inhibits PDGFR, can interrupt this paracrine loop to target KSHV tumorigenesis. Both drugs have shown efficacy in AIDS-KS clinical trials.

The remarkable oncogenic potential of KSHV conflicts with the fact that KSHV infection culminates in KS in certain epidemiological settings such as HIV infection and AIDS [2], [3]. A paradox in KSHV oncogenesis appears to be the fact that KSHV canonical latent infection cannot transform cells. In contrast, lytic infection, which expresses KSHV angiogenic genes, is cytopathic and immunogenic and, therefore, cannot transform cells in immunocompetent individuals (Figure 1) [2], [3]. Two hypotheses have been proposed: The “paracrine oncogenesis” hypothesis (Figures 1B and 2) [3] is based on the presence of lytically infected cells or latently infected cells expressing early lytic genes [27] in KS lesions. These cells express angiogenic genes, such as vGPCR, K1, and ORF45, that promote the production of angiogenic and KS growth factors (VEGF, IL-6, PDGF), which together with the virokines, stimulate the proliferation of latently infected cells and angiogenesis in a paracrine manner (Figure 2) [3], [28]. Another compatible scenario is the “abortive lytic” hypothesis (Figure 1) [22], in which cells expressing the oncogenic early lytic genes—but not the full lytic program [27]—can be transformed by genetic or epigenetic oncogenic alterations and switch back to less immunogenic latent forms, which will be paracrinally stimulated by lytically infected cells [22]. These hypotheses are supported by laboratory findings that show the paracrine nature of vGPCR-induced tumors [23], [24] and their ability to support the tumorigenicity of latent KSHV genes via paracrine mechanisms [28]. These models can explain the high incidence of KS in HIV/AIDS. Angiogenic HIV-Tat and elevated inflammatory cytokines in AIDS patients that result from chronic immune activation can induce KSHV lytic reactivation and promote KS development [29]. Furthermore, cells expressing the more immunogenic lytic genes necessary to initiate or to support KS tumors would not be eliminated in the context of AIDS immunosuppression (Figure 1) [3], [30].

## Identification and Validation of Cellular Targets Implicated in KSHV-Induced Paracrine Oncogenesis

Growth factor signaling pathways mediating KSHV-paracrine oncogenesis are attractive therapeutic targets in AIDS-KS (Figure 2). They can be targeted by FDA-approved drugs and preclinically tested in available mouse models of KSHV-induced KS [3], [5], [6], [13]. vGPCR is essential for KSHV tumorigenesis and angiogenesis [13]. vGPCR has been shown to induce KS-like tumors via the PI3K-AKT-mTOR axis by regulating expression of angiogenic growth factors as well as their paracrine activities (Figure 2) [24]. Accordingly, vGPCR tumorigenesis is blocked by the mTORC1 inhibitor rapamycin and by PI3Kγ inhibitors [24]. Furthermore, the growth of KSHV-infected xenografts was blocked by rapamycin and other rapalogs through inhibition of VEGF-angiogenesis (Figure 2) [31]. Another druggable pathway is production of reactive oxygen species (ROS) by activation of NADPH oxidase via RAC1, a downstream mediator of vGPCR-angiogenesis overexpressed in AIDS-KS lesions [20], [32]. The ROS scavenger N-Acetyl-Cysteine (NAC) was shown to inhibit KS-like tumorigenesis in a RAC1 as well as a KSHV-induced KS mouse model [20], [32]. ROS appear to play a role in paracrine oncogenesis through PDGF-mediated stimulation of cell proliferation and tumor angiogenesis mediated by STAT3 transcription of VEGF and MYC (Figure 2) [20]. Notch signaling is another important pathway activated by KSHV infection via vFLIP, vGPCR, or by regulating miR30 to promote the KS-malignant phenotype and induce angiogenesis [18], [33]. Targeting the Notch pathway with a gamma-secretase inhibitor and soluble Dll4 inhibited growth of KSHV-transformed endothelial cells in vitro and in vivo [33]. Another mediator of KSHV angiogenesis is the VEGFR2-glycan binding lectin galectin-1 [34], which is up-regulated by ROS–dependent activation of NFκB and can elicit VEGF-like signaling. A therapeutic anti-gal-1 mAb promoted KS-spindle cell xenograft regression [34].

## Translational AIDS-KS Medicine and Development of Rationally Designed Therapies

Progress in prevention and treatment of AIDS-KS is the consequence of advances in controlling HIV/AIDS as well as KSHV oncogenesis [35]. Implementation of ART led to a 6-fold decrease in AIDS-KS incidence and the regression of established AIDS-KS lesions [35], [36], while inhibition of KSHV-replication with gancyclovir decreased KS incidence in AIDS patients [37]. This is consistent with: (1) an oncogenic role of KSHV lytic replication, which is also more immunogenic and, thus, suppressed by ART-mediated immune reconstitution [30]; (2) the tumor-promoting role of HIV infection, which is targeted by ART (Figure 1B); and (3) protease inhibitors present in some ART regimens with anti-tumor, anti-angiogenic, or anti-KSHV activities [38]. AIDS-KS can present clinically as a limited, localized disease that responds to local therapies (surgical excision, cryosurgery, radiotherapy, and intralesional vinblastine) [35], [36]. AIDS-KS can also present as an aggressive, rapidly progressing, disseminated cutaneous lesion generally associated with visceral involvement that requires systemic cytotoxic chemotherapy in addition to ART [35], [36]. Although Food and Drug Administration (FDA)-approved liposomal anthracyclines (doxorubicin and daunorubicin) are effective as first-line treatment for disseminated AIDS-KS, it is estimated that more than half of these patients will not be cured [36]; therefore, new and less toxic treatment modalities are needed.

Advances in the molecular understanding of KS oncogenesis have provided a basis for the development of several targeted interventions (Figure 2) [35]. Rapamycin inhibits mTORC1, a druggable target activated by vGPCR, K1, and ORF45 [24], [25], [27], which is essential for protein translation and cell proliferation. Rapamycin was reported to cause regression of transplant-related KS [39], and its use in experimental KSHV-infected xenograft models proved to have antitumorigenic effects [31]. A recent pilot study in AIDS-KS patients showed that Rapamycin administration, in combination with ART, induced partial and total responses in some patients [40]. These responses correlated with down-regulation of mTORC1 downstream targets. Other important studies in AIDS-KS patients used Imatinib/Gleevec, which targets paracrine and autocrine tumor and angiogenic activities mediated by c-Kit and PDGFRβ (Figure 2) [41]. In this study, 50% of patients experienced a partial response that correlated with decreased phosphorylation of PDGFRβ [41]. A more recent Imatinib Phase clinical II trial showed an overall response of 33%, validating c-Kit and PDGFR as promising KS targets [42]. Other anti-angiogenic phase II trials showing clinical responses include bevacizumab/Avastin, a humanized anti-VEGF-A monoclonal antibody. These studies show that targeting paracrine mechanisms of KSHV oncogenesis is a viable approach to treat AIDS-KS patients. The continuous success of these pathogenesis-based therapies relies on understanding the close interplay between KSHV biology and the host malignant phenotype, which should inform clinical-study design and interpretation.
